# Supplementary material for: Harnessing multivalency and FcγRIIB engagement to augment anti-CD27 immunotherapy
Source: Nat Commun. 2025 Dec 20;17:1122. doi: 10.1038/s41467-025-67882-3 (PMC12855993; doi:10.1038/s41467-025-67882-3)
Supplement: Supplementary file 1 — Supplementary Information [file 41467_2025_67882_MOESM1_ESM.pdf]

Supplementary Information

Harnessing Multivalency and FcγRIIB Engagement to Augment Anti-CD27 Immunotherapy.

Marcus A. Widdess[1], Anastasia Pakidi[1], Hannah J. Metcalfe[1], H.T. Claude Chan[1], Tatyana Inzhelevskaya[1], Chris A Penfold[1], C. Ian Mockridge[1], Steven G. Booth[1], Sonya James[1], Sean H. Lim[1], Stephen A. Beers[1], Mark S. Cragg[1] and **Aymen Al-Shamkhani[1*]**.

[1]Antibody and Vaccine Group, Centre for Cancer Immunology, School of Cancer Sciences, Faculty of Medicine, University of Southampton, UK

[*]Corresponding author

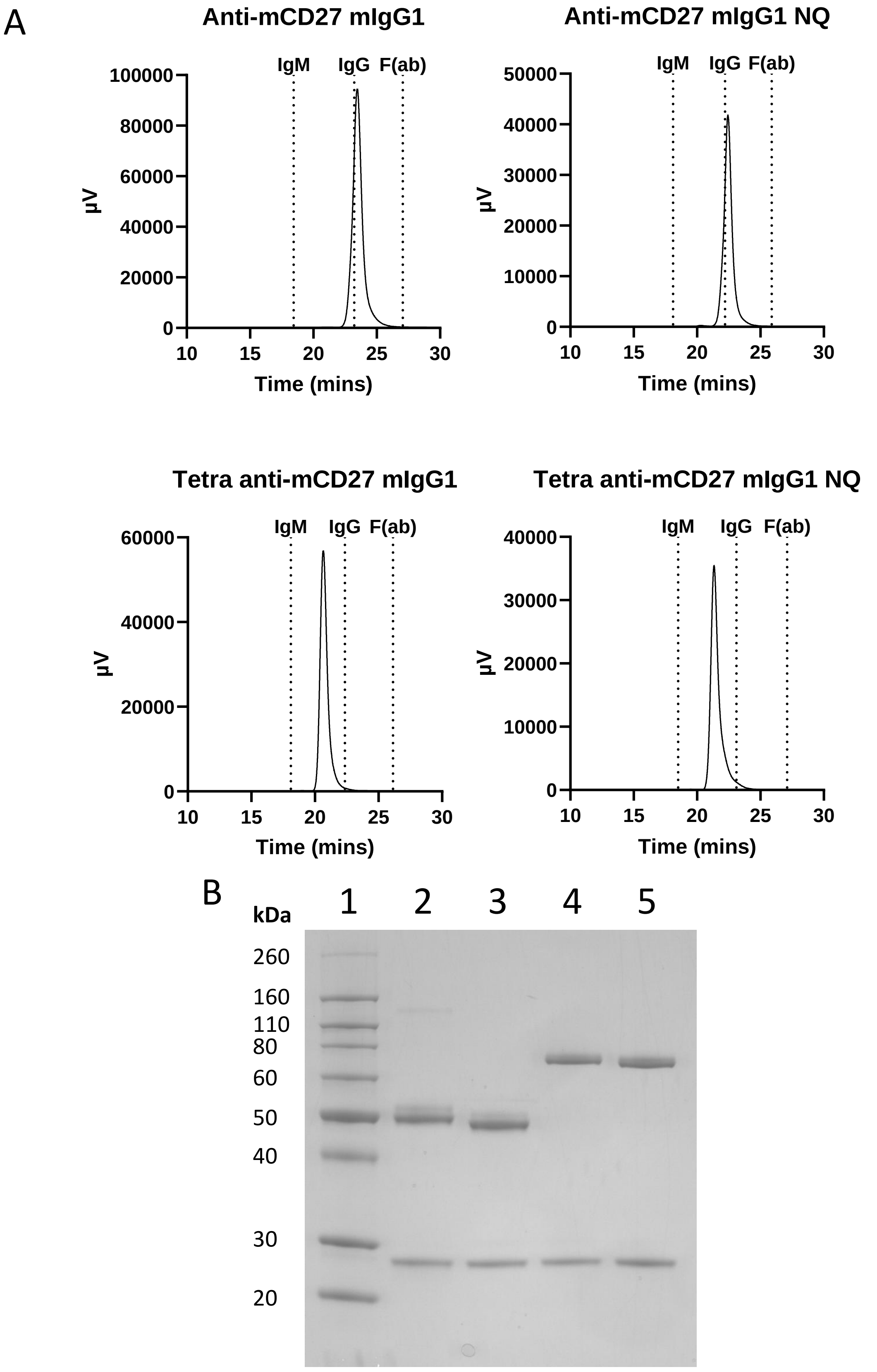

Supplementary Figure 1. Characterization of the tetravalent anti-mCD27 mIgG1 antibody by HPLC and SDS-PAGE.

a SEC traces of the indicated anti-mCD27 antibodies after production and purification, identifying a monodisperse population. Elution time of F(ab), IgG and IgM indicated by dotted lines.

b Reducing SDS-PAGE analysis of anti-mCD27 antibodies. Lanes: (1) Mw markers, (2) anti-mCD27 mIgG1, (3) anti-mCD27 mIgG1 N297Q, (4) tetra anti-mCD27 mIgG1 and (5) tetra anti-mCD27 mIgG1 N297. Mw marker/ladder band sizes indicated on the left. Source data are provided as a Source Data file.

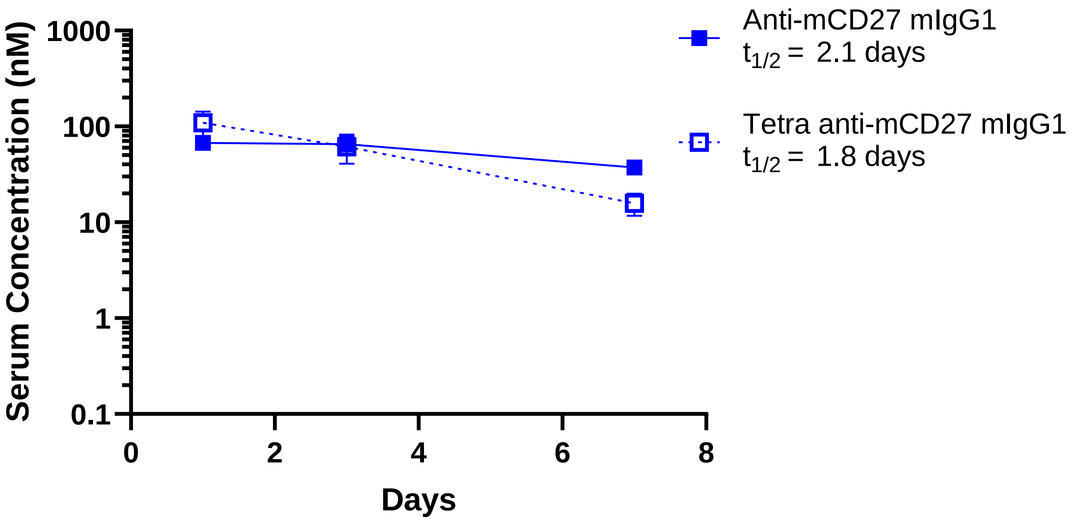

Supplementary Figure 2. Half-life analysis of the tetravalent anti-mCD27 mIgG1 antibody.
200 µg of either bivalent or tetravalent anti-mCD27 mIgG1 was intravenously injected into C57BL/6 mice. The mice were then bled at the indicated time points, with antibody concentration in plasma determined by ELISA; capturing with mCD27-ECD-his and detecting with an HRP-linked anti-mouse antibody. Data points show mean +/- SEM of 3 mice from 1 experiment. Source data are provided as a Source Data file.

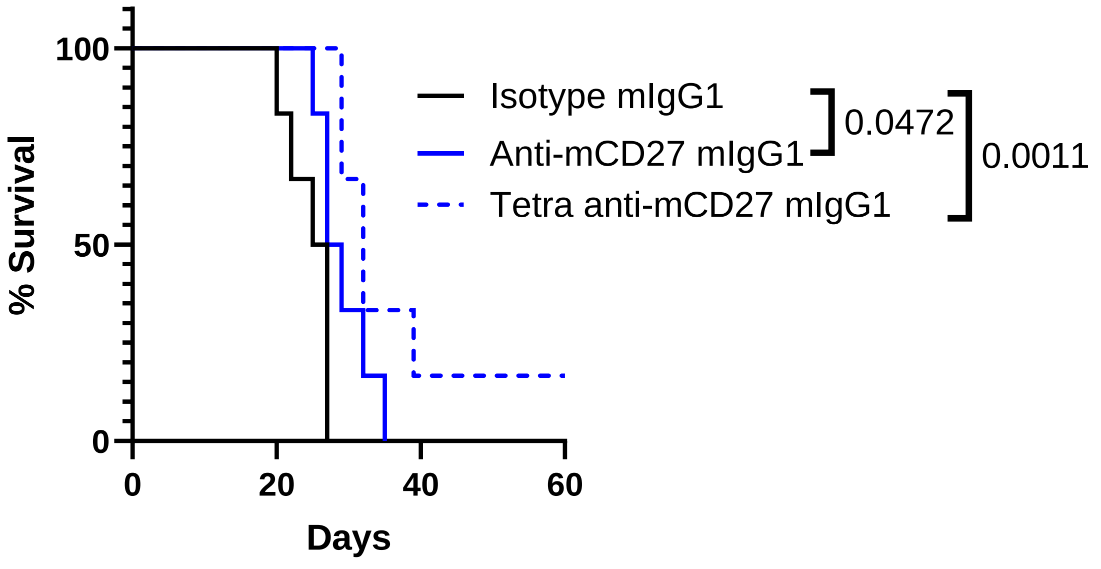

Supplementary Figure 3. The effect of anti-mCD27 antibodies on the survival of melanoma bearing mice.

Percentage survival to the humane end-point of C57BL/6 mice inoculated with B16-OVA melanoma and treated 1 day later with 1.33 nmole of the indicated antibodies + 5 mg OVA. Data shown are 6 individual mice per group from 1 experiment. Statistical significance was determined using the Log-rank (Mantel-Cox) test. Source data are provided as a Source Data file.

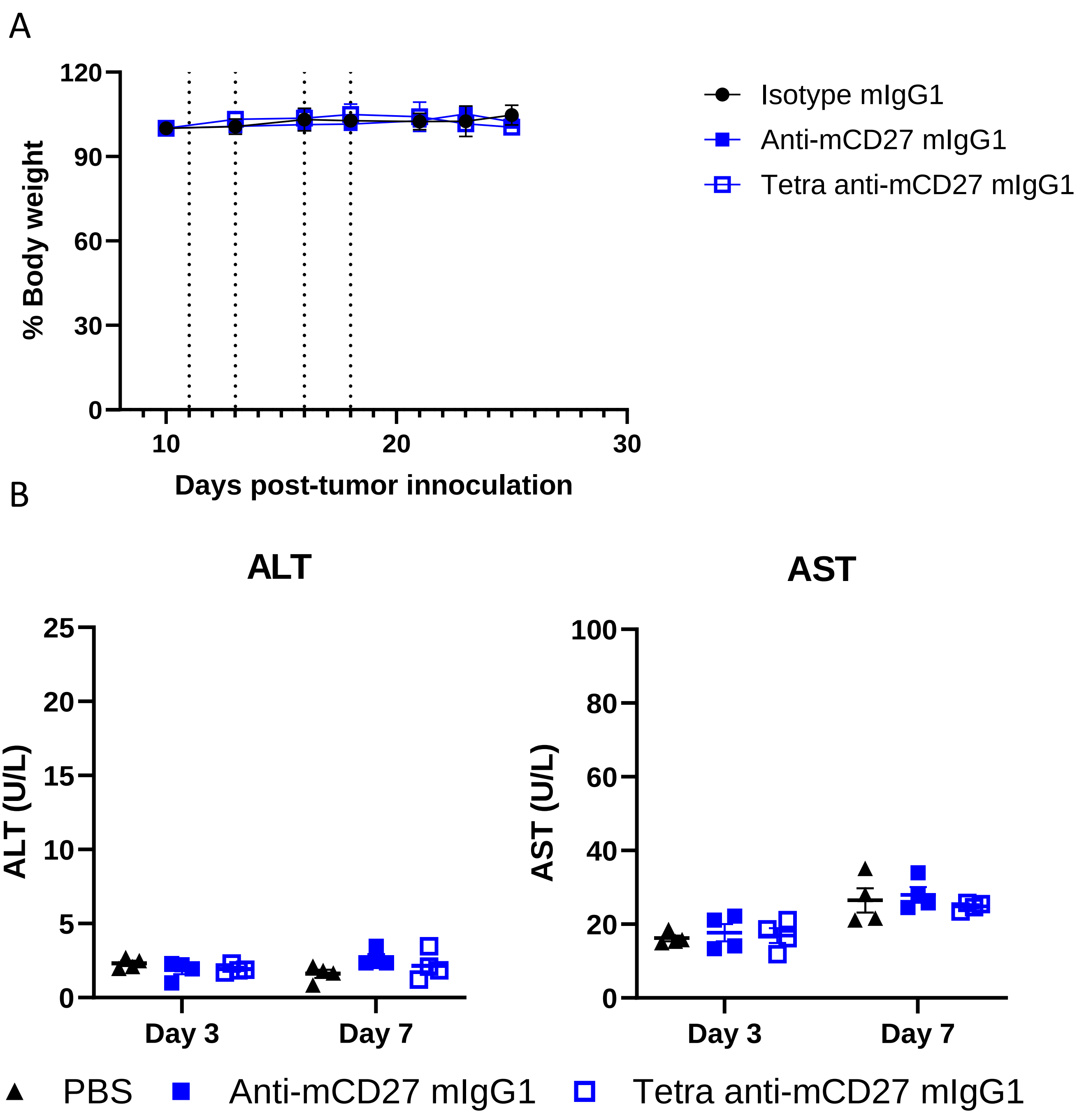

Supplementary Figure 4. Tetra anti-mCD27 mIgG1 treatment does not induce toxicity.
a Balb/c mice inoculated with CT26 colon carcinoma and treated with the indicated antibodies on days 11, 13, 16, 18 (dashed lines). Mice were weighed every 2-3 days from day 10 post tumor inoculation. Data show mean ± SEM of 5 individual mice from one experiment. **b** Balb/c mice were intraperitoneally injected with PBS or 200 µg of either anti-mCD27 mIgG1 or tetra anti-mCD27 mIgG1 on day 0. Blood sampling was performed on day 3 and day 7 and the resulting serum was assessed for ALT (left) and AST (right) activity. Data represent mean ± SEM for 4 individual mice from 1 experiment. Source data are provided as a Source Data file.

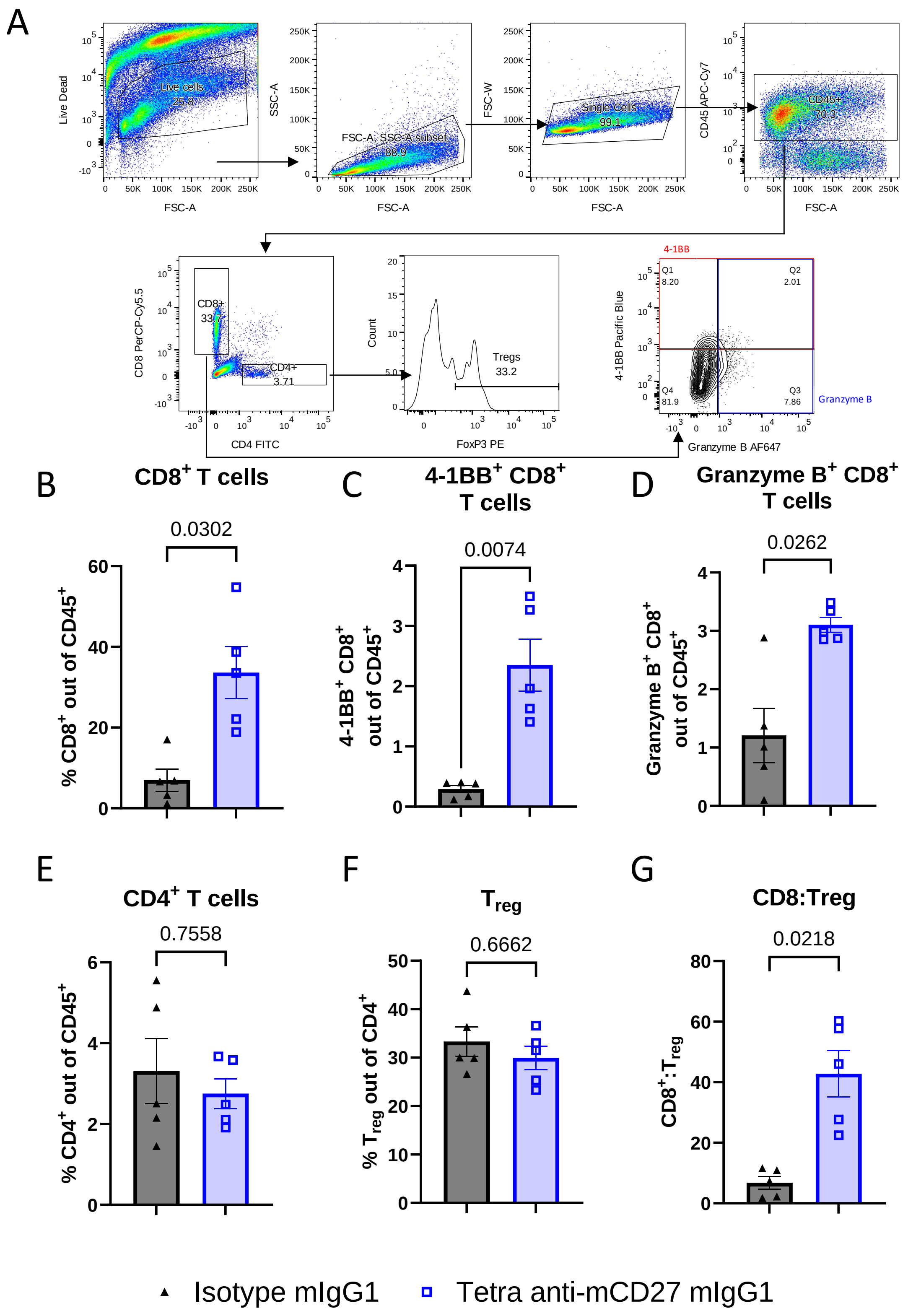

Supplementary Figure 5. Tumor infiltrating lymphocyte subsets in CT26-bearing mice. CT26 tumors were inoculated into Balb/c mice, before treatment with 100 µg of either isotype mIgG1 or tetra anti-mCD27 mIgG1 on days 11 and 13. Tumors were then harvested on day 16 and stained for flow cytometry to identify tumor infiltrating lymphocytes. **a** gating strategy used to analyse tumour infiltrating lymphocytes. **b-g** Proportion of **b** $CD8^+$ T cells out of $CD45^+$ cells, **c** 4-$1BB^+$ $CD8^+$ T cells out of $CD45^+$ cells, **d** Granzyme B^+ $CD8^+$ T cells out of $CD45^+$ cells, **e** $CD4^+$ T cells out of $CD45^+$ and **f** T_{reg} cells out of $CD4^+$ T cells. **g** Ratio of $CD8^+$ to T_{reg} calculated out of $CD45^+$ cells. Data show mean ± SEM for 5 mice per group. Statistical significance was determined by one-way ANOVA. Source data are provided as a Source Data file.

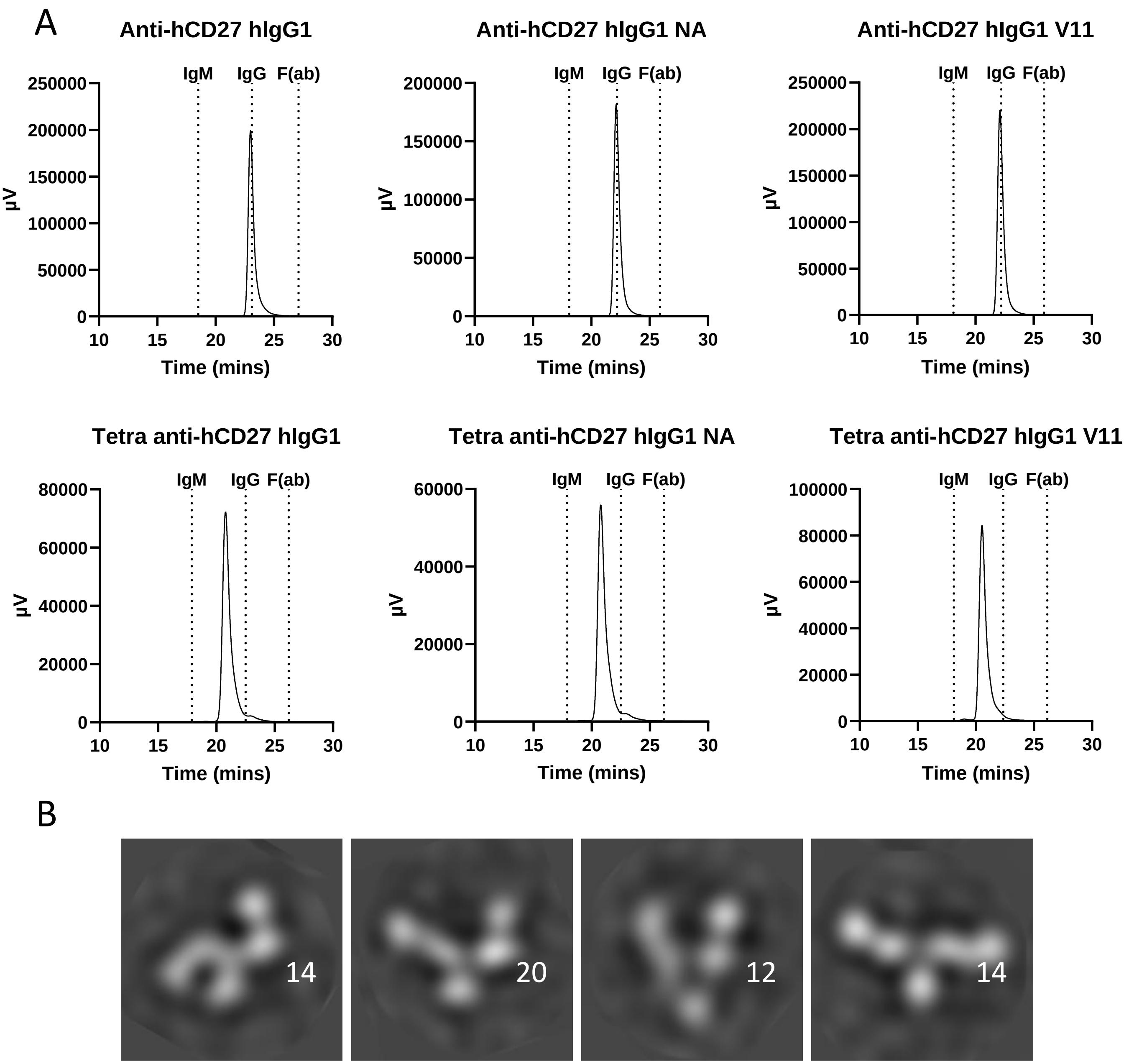

Supplementary Figure 6. Characterization of tetravalent anti-hCD27 antibodies by HPLC and negative EM.

a SEC traces of the indicated anti-hCD27 antibodies after production and purification, identifying a monodisperse population. Elution time of F(ab), IgG and IgM indicated by dotted lines. **b** Representative class averages from negative stain electron microscopy of Tetra anti-hCD27 hIgG1 V11. Source data are provided as a Source Data file.

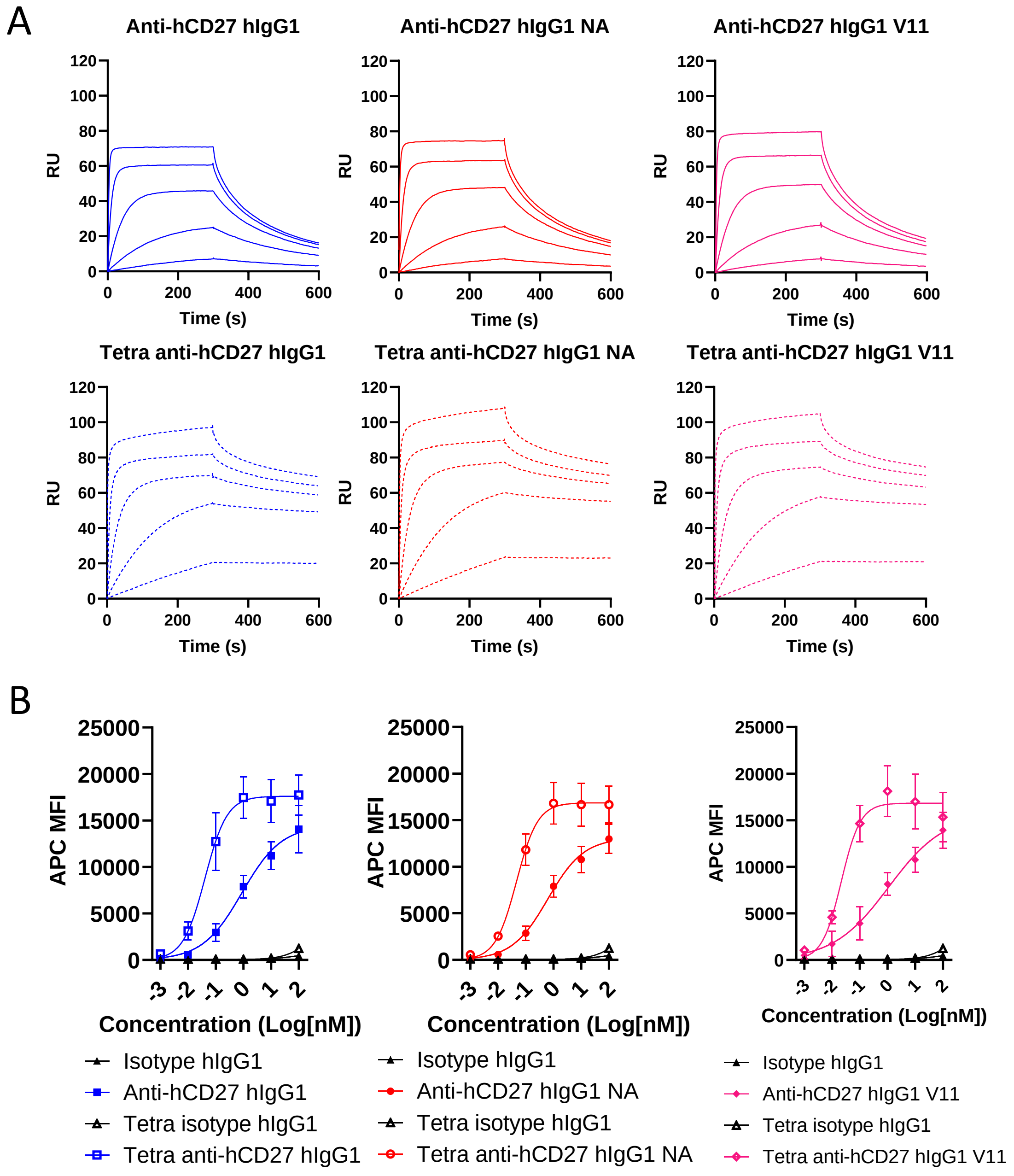

Supplementary Figure 7. Tetravalency enhances anti-hCD27 avidity for hCD27.
a SPR analysis of tetra anti-hCD27 antibodies. The indicated antibodies were injected over immobilized hCD27-Fc for 300s at a starting concentration of 100 nM and then a 3-fold serial dilution thereof. Data show concentrations from one experiment. **b** hCD27^{+} Jurkat cells were stained with the anti-hCD27 antibodies indicated (hIgG1, left panel; hIgG1 NQ, centre panel or hIgG1 V11, right panel followed by APC-conjugated anti-human Fc secondary F(ab)$_2$. Secondary antibody binding was detected by flow cytometry. Data show mean ± SEM from 3 experiments. Source data are provided as a Source Data file.

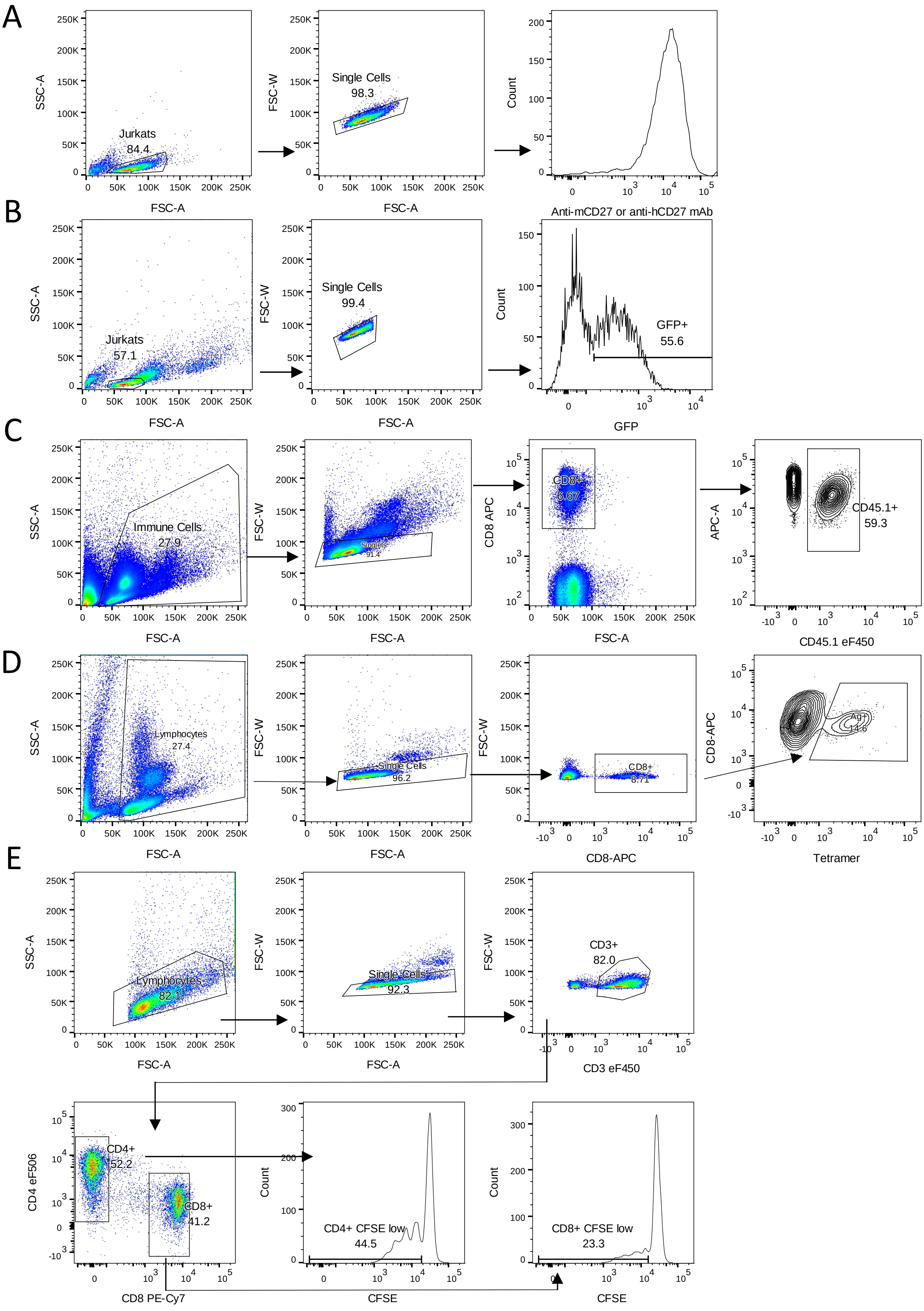

Supplementary Figure 8. Gating strategies for flow cytometry.
a gating strategy to assess anti-CD27 antibody binding to Jurkat mCD27⁺ or Jurkat hCD27⁺ cells in Figure 1c or supplementary Figure 7b. **b** gating strategy to assess GFP production in NF-κB-GFP Jurkat cells expressing either mCD27, **i**n Figure 2a-b, or hCD27, in Figure 6a. **c** gating strategy to identify CD8⁺ CD45.1⁺ cells in blood samples from **F**igure 3a-c. **d** gating strategy to identify CD8⁺ Tetramer⁺ cells in blood samples from Figure 4a-c. **e** gating strategy to identify CD4⁺ CFSE low and CD8⁺ CFSE low cells from Figure 6b. Source data are provided as a Source Data file.

Antibody	Melting temperature (°C)		
	Run 1	Run 2	Run 3
Anti-hCD27 hIgG1 V11	63.5	63.5	63.6
Tetravalent anti-hCD27 hIgG1 V11	56.6	56.3	57.2

Supplementary Table 1. Summary of melting temperatures of anti-hCD27 hIgG1 V11 antibodies determined by Nano differential scanning fluorimetry.

NanoDSF was used to assess thermal stability of tetravalent anti-hCD27 hIgG1 V11; with the data shown as technical replicates from one experiment. Source data are provided as a Source Data file.

Antibody	K_a (x 10^6 $M^{-1}s^{-1}$)	K_d (x 10^{-3} s^{-1})	Apparent affinity (Kd, x 10^{-9} M)
Anti-hCD27 hIgG1	3.1	9.7	3.1
Anti-hCD27 hIgG1 NA	2.9	9.4	3.1
Anti-hCD27 hIgG1 V11	2.6	9.9	3.7
Tetravalent anti-hCD27 hIgG1	2.0	2.8	1.4
Tetravalent anti-hCD27 hIgG1 NA	1.9	2.8	1.5
Tetravalent anti-hCD27 hIgG1 V11	2.1	2.8	1.4

Supplementary Table 2. Summary of binding kinetics of anti-hCD27 antibodies.
Kinetic fits of anti-hCD27 antibody binding were calculated from SPR analysis in Fig. S7 using the Biacore kinetics summary v3.1 software and the bivalent (2:1) binding model. Data from one experiment. Source data are provided as a Source Data file.

Antibody	Clone	Isotype	Label	Company	Code	Dilution
Anti-mCD8α	53-6.7	rIgG2a	APC	eBioscience	17-0081-82	1:400
Anti-mCD8α	53-6.7	rIgG2a	PerCP-Cy5.5	eBioscience	45-0081-82	1:200
Anti-mFOXP3	FJK-16s	rIgG2a	PE	eBioscience	12-5773-82	1:200
Anti-CD45.1	A20	mIgG2a	eFluor (eF) 450	eBioscience	48-0453-82	1:100
Anti-mCD4	GK1.5	rIgG2b	FITC	eBioscience	11-0041-82	1:100
Anti-hCD3e	UCHT1	mIgG1	eF450	eBioscience	48-0038-82	1:100
Anti-hCD8a	RPA-T8	mIgG1	PE-Cy7	eBioscience	25-0088-42	1:100
Anti-m4-1BB	17B5	Syrian Hamster IgG	eF450	eBioscience	48-1371-82	1:100
Anti-hCD4	RPA-T4	mIgG1	eF506	eBioscience	69-0049-42	1:100
Anti-mouse granzyme B	GB11	mIgG1	Alexa Fluor (AF) 647	Biolegend	515406	1:100
Anti-mCD45.2	104	mIgG2a	APC-eF780	eBioscience	47-0454-82	1:100
AffiniPure Anti-Mouse IgG, Fcγ fragment specific	Polyclonal	Goat F(ab')2	APC	Jackson ImmunoResearch	115-136-071	1:400
AffiniPure Anti-human IgG, Fcγ fragment specific	Polyclonal	Goat F(ab')2	APC	Jackson ImmunoResearch	109-136-170	1:400
AffiniPure $F(ab')_2$ Fragment Donkey Anti-Human IgG (H+L)	Polyclonal	Donkey F(ab')2	Unlabelled	Jackson ImmunoResearch	709-006-149	1:500

Supplementary Table 3. Summary of commercial antibodies.
A summary of commercial antibodies used, with dilutions.

Antibody	Clone	Isotype	Dilution
Isotype	AT171-2	mIgG1	Indicated in figure.
Anti-mCD27	AT124-1	mIgG1	Indicated in figure.
Anti-mCD27	AT124-1	mIgG1 NQ	Indicated in figure.
Tetra isotype	AT171-2	mIgG1	Indicated in figure.
Tetra anti-mCD27	AT124-1	mIgG1	Indicated in figure.
Tetra anti-mCD27	AT124-1	mIgG1 NQ	Indicated in figure.
Isotype	AT171-2	hIgG1	Indicated in figure.
Anti-hCD27	hCD27.15	hIgG1	Indicated in figure.
Anti-hCD27	hCD27.15	hIgG1 NA	Indicated in figure.
Anti-hCD27	hCD27.15	hIgG1 V11	Indicated in figure.
Tetra isotype	AT171-2	hIgG1	Indicated in figure.
Tetra anti-hCD27	hCD27.15	hIgG1	Indicated in figure.
Tetra anti-hCD27	hCD27.15	hIgG1 NA	Indicated in figure.
Tetra anti-hCD27	hCD27.15	hIgG1 V11	Indicated in figure.
R-Phycoerythrin-conjugated tetrameric H2Kb-OVA(257-264)	-	-	1:200

Supplementary Table 4. Summary of in-house antibodies.
A summary of in-house produced antibodies.
