# Supplementary material for: Harnessing multivalency and FcγRIIB engagement to augment anti-CD27 immunotherapy
Source: Nat Commun. 2025 Dec 20;17:1122. doi: 10.1038/s41467-025-67882-3 (PMC12855993; doi:10.1038/s41467-025-67882-3)
Supplement: Supplementary file 2 — Reporting Summary [file 41467_2025_67882_MOESM2_ESM.pdf]

nature portfolio

Corresponding author(s): Aymen Al-Shamkhani

Last updated by author(s): Dec 10, 2025

Reporting Summary

Nature Portfolio wishes to improve the reproducibility of the work that we publish. This form provides structure for consistency and transparency in reporting. For further information on Nature Portfolio policies, see our Editorial Policies and the Editorial Policy Checklist.

Statistics

For all statistical analyses, confirm that the following items are present in the figure legend, table legend, main text, or Methods section.

n/a	Confirmed	
☐	☒	The exact sample size (*n*) for each experimental group/condition, given as a discrete number and unit of measurement
☐	☒	A statement on whether measurements were taken from distinct samples or whether the same sample was measured repeatedly
☐	☒	The statistical test(s) used AND whether they are one- or two-sided<br>*Only common tests should be described solely by name; describe more complex techniques in the Methods section.*
☒	☐	A description of all covariates tested
☐	☒	A description of any assumptions or corrections, such as tests of normality and adjustment for multiple comparisons
☐	☒	A full description of the statistical parameters including central tendency (e.g. means) or other basic estimates (e.g. regression coefficient) AND variation (e.g. standard deviation) or associated estimates of uncertainty (e.g. confidence intervals)
☐	☒	For null hypothesis testing, the test statistic (e.g. *F*, *t*, *r*) with confidence intervals, effect sizes, degrees of freedom and *P* value noted<br>*Give P values as exact values whenever suitable.*
☒	☐	For Bayesian analysis, information on the choice of priors and Markov chain Monte Carlo settings
☒	☐	For hierarchical and complex designs, identification of the appropriate level for tests and full reporting of outcomes
☒	☐	Estimates of effect sizes (e.g. Cohen's *d*, Pearson's *r*), indicating how they were calculated

Our web collection on statistics for biologists contains articles on many of the points above.

Software and code

Policy information about availability of computer code

Data collection	Flow cytometry data: BD FACSDiva software (Version 9), BD CellQuest software (Version 6.1).<br>Negative stain EM: RELION v3.1.3 (Sjors Scheres, MRC Laboratory of Molecular Biology).<br>Confocal images: NimOS v1.18.<br>Biacore T200 control software (Version 2.0.2) was used to collect SPR data
Data analysis	Biacore T200 Evaluation software (Version 3.1) was used to analyse SPR data<br>GraphPad Prism (Version 10.0.3) was used for data analysis and to perform statistical tests<br>FlowJo (Version 10.8.1) was used to analyse flow cytometry data<br>Images were analysed by NimOS v1.18 and ImageJ v1.53k<br>ThermControl software (NanoTemperTechnologies) first derivative analysis of nano differential scanning fluorimetry (NanoDSF) data

For manuscripts utilizing custom algorithms or software that are central to the research but not yet described in published literature, software must be made available to editors and reviewers. We strongly encourage code deposition in a community repository (e.g. GitHub). See the Nature Portfolio guidelines for submitting code & software for further information.

Data

Policy information about availability of data

All manuscripts must include a data availability statement. This statement should provide the following information, where applicable:

- Accession codes, unique identifiers, or web links for publicly available datasets
- A description of any restrictions on data availability
- For clinical datasets or third party data, please ensure that the statement adheres to our policy

All data are included in the Supplementary Information or available from the authors, as are unique reagents used in this Article. The raw numbers for charts and graphs are available in the Source Data file whenever possible.
Correspondence and material requests should be addressed to Prof Aymen Al-Shamkhani. Email: aymen@soton.ac.uk.

Research involving human participants, their data, or biological material

Policy information about studies with human participants or human data. See also policy information about sex, gender (identity/presentation), and sexual orientation and race, ethnicity and racism.

Reporting on sex and gender	N/A
Reporting on race, ethnicity, or other socially relevant groupings	N/A
Population characteristics	No human research participants were engaged directly during the study. Biospecimens were obtained from anonymous healthy donors providing blood donations to the NHS blood transfusion service. Samples were derived from both males and females.
Recruitment	Anonymous donors provide blood to the NHS blood transfusion service with secondary blood products provided for use to ethically approved studies with prior informed consent.
Ethics oversight	The use of human tissue was approved by the East of Scotland Research Ethics Service, Tayside, UK and via the Faculty of Medicine Research Ethics Committee under submission 19660.

Note that full information on the approval of the study protocol must also be provided in the manuscript.

Field-specific reporting

Please select the one below that is the best fit for your research. If you are not sure, read the appropriate sections before making your selection.

☒ Life sciences ☐ Behavioural & social sciences ☐ Ecological, evolutionary & environmental sciences

For a reference copy of the document with all sections, see nature.com/documents/nr-reporting-summary-flat.pdf

Life sciences study design

All studies must disclose on these points even when the disclosure is negative.

Sample size	Sample sizes were chosen based upon prior experience of numbers required to demonstrate statistical significance in similar prior studies. For immunotherapy experiments we estimate that we need to be able to detect the difference between a control group with a median survival of 12 days and a test group with a median survival of 30 days. A power analysis calculation based at the 5% significance level and with 80% power predicts 19 animals per group. However, we are using inbred strains of mice, so intra-group variability is reduced. From experience, we have found that we can use considerably fewer animals per group, usually 5, to detect such a difference at the 5% significance level in the EG7 model. For monitoring immunological responses we typically use 3-5 mice per group for each experiment. From our experience, this number of mice/group allows us to identify, with a statistical power of 95%, a 3-fold difference in the number of CD8 T cells between 2 groups at the 5% significance level.
Data exclusions	No data was excluded from the analysis.
Replication	All mouse tumor experiments were repeated at least twice, and all in vitro experiments were repeated 2 or more times. Details of experimental replicates are stated in the figure legends. All attempts at replication were successful.
Randomization	Animals were randomly divided into groups based on age and gender. Allocation of samples into different treatment groups was random or not relevant (e.g. where cells from a single sample was treated with multiple different mAb).
Blinding	Blinding was not performed due to limited numbers of experienced personnel associated with this research project.

Reporting for specific materials, systems and methods

We require information from authors about some types of materials, experimental systems and methods used in many studies. Here, indicate whether each material, system or method listed is relevant to your study. If you are not sure if a list item applies to your research, read the appropriate section before selecting a response.

Materials & experimental systems

n/a	Involved in the study
[ ]	[x] Antibodies
[ ]	[x] Eukaryotic cell lines
[x]	[ ] Palaeontology and archaeology
[ ]	[x] Animals and other organisms
[x]	[ ] Clinical data
[x]	[ ] Dual use research of concern
[x]	[ ] Plants

Methods

n/a	Involved in the study
[x]	[ ] ChIP-seq
[ ]	[x] Flow cytometry
[x]	[ ] MRI-based neuroimaging

Antibodies

Antibodies used

In house antibodies:
Isotype mIgG1, clone AT171-2, dilution in figure.
Anti-mCD27 mIgG1, clone AT124-1, dilution in figure.
Anti-mCD27 mIgG1 N297Q, clone AT124-1, dilution in figure.
Tetravalent isotype mIgG1, clone AT171-2, dilution in figure.
Tetravalent anti-mCD27 mIgG1, clone AT124-1, dilution in figure.
Tetravalent anti-mCD27 mIgG1 N297Q, clone AT124-1, dilution in figure.
Isotype hIgG1, clone AT171-2, dilution in figure.
Anti-hCD27 hIgG1, clone hCD27.15, dilution in figure.
Anti-hCD27 hIgG1 N297A, clone hCD27.15, dilution in figure.
Anti-hCD27 hIgG1 V11, clone hCD27.15, dilution in figure.
Tetravalent anti-hCD27 hIgG1, clone hCD27.15, dilution in figure.
Tetravalent anti-hCD27 hIgG1 N297A, clone hCD27.15, dilution in figure.
Tetravalent anti-hCD27 hhgG1 V11, clone hCD27.15, dilution in figure.
R-Phycoerythrin-conjugated tetrameric H2Kb-OVA(257-264) peptide, 1:200.
Clone AT124-1 validation: (https://doi.org/10.1182/blood-2006-11-057216)
Clone hCD27.15 validation: (https://doi.org/10.1038/s42003-022-03182-6)

Commercial antibodies
Anti-mCD8α, 53-6.7, rIgG2a, APC, eBioscience, 17-0081-82, RRID:AB_469335, 1:400
Anti-mCD8α, 53-6.7, rIgG2a, PerCP-Cy5.5, eBioscience, 45-0081-82, RRID:AB_1107004, 1:200
Anti-mFOXP3, FJK-16s, rIgG2a, PE, eBioscience, 12-5773-82, RRID:AB_465936, 1:200
Anti-CD45.1, A20, mIgG2a, eFluor (eF) 450, eBioscience, 48-0453-82, RRID:AB_1272189, 1:100
Anti-mCD4, GK1.5, rIgG2b, FITC, eBioscience, 11-0041-82, RRID:AB_464892, 1:100
Anti-hCD3e, UCHT1, mIgG1, eF450, eBioscience, 48-0038-82, RRID:AB_1518799, 1:100
Anti-hCD8a, RPA-T8, mIgG1, PE-Cy7, eBioscience, 25-0088-42, RRID:AB_1659702, 1:100
Anti-m4-1BB, 17B5, Syrian Hamster IgG, eF450, eBioscience, 48-1371-82, RRID:AB_2574041, 1:100
Anti-hCD4, RPA-T4, mIgG1, eF506, eBioscience, 69-0049-42, RRID:AB_2637466, 1:100
Anti-mouse granzyme B, GB11, mIgG1, Alexa Fluor (AF) 647, Biolegend, 515406, AB_2566333, 1:100
Anti-mCD45.2, 104, mIgG2a, APC-eF780, eBioscience, 47-0454-82, RRID:AB_1272175, 1:100
AffiniPure Anti-Mouse IgG, Fcγ fragment specific, Polyclonal, Goat F(ab')2, APC, Jackson ImmunoResearch, 115-136-071, RRID:AB_2338648, 1:400
AffiniPure Anti-human IgG, Fcγ fragment specific, Polyclonal, Goat F(ab')2, APC, Jackson ImmunoResearch, 109-136-170, RRID:AB_2337695, 1:400
AffiniPure F(ab')$_2$ Fragment Donkey Anti-Human IgG (H+L), Polyclonal, Donkey F(ab')2, Unlabelled, Jackson ImmunoResearch, 709-006-149, RRID:AB_2340486, 1:500

Validation

The specificity of commercial antibodies was verified by the manufacturer, in-house antibodies were verified using cell lines specific to their species specificity and data in this manuscript support their specificity. All in-house antibodies are subjected to rigorous QC including being checked by HPLC to contain < 1% aggregate and by Endosafe-PTS portable test (Charles River Laboratories, L'Arbresle, France) to contain < 5EU endotoxin/mg antibody.

Eukaryotic cell lines

Policy information about cell lines and Sex and Gender in Research

Cell line source(s)

Jurkat NF-κB reporter T cell line (Jurkat NF-κB GFP, System Biosciences, TR850A-1), CHO-k1 (ATCC, CCL-61), CT26 colon carcinoma (ATCC, CRL-2638).

	BCL1 validated by idiotype (J Immunol 1979 Vol. 123 Issue 3 Pages 1181-8). B16-OVA-GFP generated by transduction of B16 melanoma cells originally from ATCC (J Clin Invest 2008 Vol. 118 Issue 6 Pages 2098-110 provided by Professor Caetano Reis e Sousa, The Francis Crick Institute, UK) validated immunologically and by GFP expression.
Authentication	For cell lines purchased from ATCC or commercial providers (System biosciences) further authentication was not performed given clear provenance. BCL1 cells were validated by idiotype. B16-OVA-GFP were validated immunologically and by GFP expression.
Mycoplasma contamination	Mycoplasma test were conducted using the Mycoplasma: MycoAlert Mycoplasma Detection Kit (Lonza) and returned negative results.
Commonly misidentified lines (See ICLAC register)	None were used in this study

Animals and other research organisms

Policy information about studies involving animals; ARRIVE guidelines recommended for reporting animal research, and Sex and Gender in Research

Laboratory animals	C57BL/6J (Charles River UK, Strain code: 027), Balb/c (Strain code: 028, Charles River UK) and OT-I transgenic (Tg) mice (Charles River France, strain code: 642), were purchased from Charles River and stock colonies maintained by the University of Southampton Biomedical Research Facility. For C57BL/6 mice, 54 female 8-12 week old mice were used. For Balb/c mice, 81 female 8-12 week old mice were used. For OT-I Tg mice, 20 female 8-12 week old mice were used. All mice were randomly assigned into experimental groups, with experimental and control animals co-housed. Mice were maintained on a 12-hour light and dark cycle, an ambient temperature of 20-24 °C, 55 % humidity ± 15 %, with food and water ad libitum. Mice were kept under specific pathogen free (SPF) conditions. Daily checks were performed to ensure mice remained healthy and environmental enrichment was provided. Mice were euthanised by CO2 inhalation or cervical dislocation.
Wild animals	No wild animals were used in this study.
Reporting on sex	Sex does not impact the results of this study.
Field-collected samples	No field collected samples were used in this study.
Ethics oversight	All experiments were conducted following University of Southampton ethical approval and in accordance with the Animals (Scientific Procedures) Act 1986 as set out in PPL: P4D9C89EA and PIL: I66C5D543

Note that full information on the approval of the study protocol must also be provided in the manuscript.

Plants

Seed stocks	Plants were not used in this study.
Novel plant genotypes	Plants were not used in this study.
Authentication	Plants were not used in this study.

Flow Cytometry

Plots

Confirm that:

- [x] The axis labels state the marker and fluorochrome used (e.g. CD4-FITC).
- [x] The axis scales are clearly visible. Include numbers along axes only for bottom left plot of group (a 'group' is an analysis of identical markers).
- [x] All plots are contour plots with outliers or pseudocolor plots.
- [x] A numerical value for number of cells or percentage (with statistics) is provided.

Methodology

Sample preparation	Cells were prepared for flow cytometry by centrifugation at 450 g for 5 mins and resuspended in PBS
Instrument	Flow cytometry was performed using either a FACS Calibur or FACS Canto II (BD Biosciences).
Software	FACS Calibur data were collected using BD Cell Quest and FACS Canto II data were collected using BD FACSDIVA. Data were

	analysed using FlowJo.
Cell population abundance	For experiments, 5,000 - 10,000 cells were collected, and samples were gated based on their FSC/SSC properties.
Gating strategy	FSC/SCC gates were based on prior knowledge of the position of viable cells in a population based on these parameters due to the cell size and granularity. Positive cell populations were selected based on the observation of distinct populations that were stained for the cell marker being probed versus staining with an isotype control. No complex gating was required and so exemplar gating strategy was not included.

☒ Tick this box to confirm that a figure exemplifying the gating strategy is provided in the Supplementary Information.
